# Magnetic Resonance Imaging Artifact Elimination in the Diagnosis of Female Pelvic Abscess under Phase Correction Algorithm

**DOI:** 10.1155/2021/9873775

**Published:** 2021-07-30

**Authors:** Ying Xia, Shaozheng Chen

**Affiliations:** Department of Gynecology, Baodi Clinical College, Tianjin Medical University, Tianjin 301800, China

## Abstract

In order to explore the effect of magnetic resonance imaging (MRI) based on phase correction algorithm in diagnosing female pelvic abscess, firstly, the effect of phase correction algorithm on eliminating MRI image motion artifacts was studied, then it was applied to 71 female pelvic cases admitted to our hospital in the diagnosis of abscess patients with magnetic resonance imaging technology, and the results were compared with the results of multislice spiral CT and laparoscopy to explore the accuracy of MRI and CT. It was found that the results of MRI examination were close to those of laparoscopy, and the difference was not statistically significant (*P* > 0.05); the results of CT examination and laparoscopy were significantly different, and the difference was statistically significant (*P* < 0.05); in addition, the results of CT examination, the number of bacterial cysts (43 cases) and tuberculous cysts (12 cases), were significantly lower than the results of MRI (50 cases, 18 cases), and the difference was statistically significant (*P* < 0.05). The size of the mass shown by the MRI examination (4.1  cm × 4.2  cm × 3.9 cm～13.9  cm × 9.5  cm × 8.7 cm) was larger than the size of the mass revealed by the CT examination (5.2  cm × 4.3  cm × 4.1 cm～15.5  cm × 10.1  cm × 9.6 cm), the difference between the two was statistically significant (*P* < 0.05), and it was closer to the results of laparoscopic pathology (4.1  cm × 4.3  cm × 3.9 cm～14.1  cm × 9.3  cm*P* < 0.058.7 cm). In short, the phase correction algorithm could eliminate the motion artifacts of MRI images. In the imaging diagnosis of female pelvic abscess, the MRI diagnosis based on the phase correction algorithm is more ideal than the diagnosis of multislice spiral CT. It can be used as a reference basis for clinical disease treatment.

## 1. Introduction

Pelvic abscess is a common disease in gynecology [1]. Its main causes include *Chlamydia trachomatis* and *Neisseria gonorrhoeae* infection; and its high-risk factors include abnormal sexual intercourse, unreasonable use of drugs, neglect of hygiene during menstruation, postpartum, miscarriage, or postoperative bacterial infection, and inflammatory reaction of nearby organs [2, 3]. In some patients with pelvic abscess, the inflammatory reaction only affects a fixed part, while the inflammatory reaction may be involved in multiple adjacent parts for some other patients. The pathogenesis generally starts from inflammatory infiltration and progresses to tissue edema or adhesion. An inflammatory mass is manifested in different sizes and shapes, which seriously affects the work, study, and life of patients [4]. If pelvic abscess is not properly and thoroughly treated, it will progress to nephropathy and sepsis, and even septic shock, endangering the life of the patient [5]. Pelvic abscess has to be diagnosed by the patient in cooperation with the doctor's examination. When the pelvic abscess is examined through medical imaging, it is very dependent on the resolution and clarity of the imaging. Therefore, doctors have to be proficient in the imaging of pelvic abscess, which is helpful for correct clinical diagnosis and selection of the best timing of surgery [6].

With the advancement of science and technology, MRI technology is increasingly used in medical diagnosis. As a multiparameter imaging, MRI uses the magnetic resonance to generate magnetic resonance signals to reconstruct human body information, thereby forming images, which can provide richer information and can be viewed from any angle, such as cross section, sagittal plane, coronal plane, and various oblique planes, providing a more detailed anatomical Atlas [7]. In the study of pelvic abscess, Meller et al. [8] believed that ultrasound, CT, and other inspection methods did not have advantages over MRI. Although CT is relatively simple to operate and less expensive for patients, it had higher requirements on the examiner and requires certain operating experience, and the diagnostic parameters of ultrasound and CT were less. The patient data obtained are not enough to support the diagnosis. The diagnosis and treatment are prone to adverse effects. The resolution of MRI images is relatively clear, the images can be observed in multiple directions, and the soft tissue can be visually compared and analyzed. In addition, it can show the anatomical relationship among the surrounding organs and tissues of the pelvis, distinguishing the characteristics of FPA in all directions, causing no radioactive damage, and showing little side effects [9].

Since MRI imaging takes longer time than other inspection methods, and it is more susceptible to various voluntary or involuntary movements of the patient, causing image artifacts, reducing the image quality of MRI imaging, and seriously interfering with clinical diagnosis [10]. Scholars have conducted a lot of research and treatment in this situation [11]. The methods that can effectively reduce motion artifacts and improve imaging quality are classified into two categories. One is to minimize the patient's autonomous or involuntary movement and reduce the effect to imaging; and the other is to use a method based on signal processing to restore the damaged image data, which is more effective for some images that cannot be removed by the first method [12, 13]. Research findings confirm that the correction algorithm can effectively eliminate artifacts in MRI images. This study first explored the effect of eliminating MRI image motion artifacts based on the phase correction algorithm, then applied it to the diagnosis of female pelvic abscess patients with MRI technology, and compared it with the results of multislice spiral CT and laparoscopy, so as to explore the accuracy of MRI and CT inspections, hoping to provide some theoretical references for improving the accuracy and efficiency of the diagnosis of pelvic abscess disease.

## 2. Materials and Methods

### 2.1. Objects

71 FPA patients admitted to the hospital from January 2018 to December 2020 were selected. The patients were aged 20–68 years, with an average age of 38.14 ± 6.43 years. The main clinical manifestations were high fever, vaginal bleeding, lower abdominal pain, nausea and vomiting, and physical fatigue (Table 1). The abovementioned clinical manifestations were overlapping. The gap in general information of all patients was not statistically obvious (*P* > 0.05), and there was comparability. CT and MRI examinations were performed on all patients. The experimental procedure had been approved by the ethics committee of hospital, and all subjects included in the study had signed the informed consent forms.

### 2.2. Establishment of an MRI Detection Model Based on Phase Correction Algorithm

MRI imaging technology was to store the original data obtained by scanning according to a unique method. Scholars named the space for storing the original data as K-space. The K-space data *S (k*_*x*_*, k*_*y*_) were the frequency-domain information of the imaged object *m (x, y)*, as shown in the following equation:

(1) $$\begin{array}{c} Sk_{x},k_{y}=\iint m\left(x,y\right)e^{-i2\pi \left(k_{x}x+k_{y}y\right)}\text{d}x\text{d}y. \end{array}$$

When the data were obtained by scanning, the movement distances of the target in the *x-* and *y*-direction were set as *p* (*k*_*y*_) and *q* (*k*_*y*_), respectively, and the signal could be obtained based on the following equation:

(2) $$\begin{array}{c} S^{\prime }\left(k_{x},k_{y}\right)=\iint m\left(x-p\left(k_{y}\right),y-q\left(k_{y}\right)\right)e^{-i2\pi \left(k_{x}x+k_{y}y\right)}\text{d}x\text{d}y,\\ S^{\prime }\left(k_{x},k_{y}\right)=e^{-i\phi \left(k_{x},k_{y}\right)}S\left(k_{x},k_{y}\right). \end{array}$$

In the abovementioned equation, *ϕ*(*k*_*x*_*, k*_*y*_) met *ϕ*(*k*_*x*_, *k*_*y*_) *=* 2*π*(*k*_*x*_*p*(*k*_*y*_)*+k*_*x*_*q*(*k*_*y*_)). Because the scan time of MRI was long, the patient was prone to autonomous or involuntary movement, which caused the data to shift in the *x-* and *y*-direction. To eliminate the phase difference between the original data and the artifact data in the K-space, it was necessary to correct the K-space with some measures and further extract the interest area. The calculation method was as follows:

(3) $$\begin{array}{c} E_{\text{snake}}\left(V\right)=E_{\mathrm{int}}\left(V\right)+E_{\text{ext}}\left(V\right). \end{array}$$

In the abovementioned equation, *E*_int_(*V*) represents the internal energy of the curve shape and *E*_ext_(*V*) represents the external energy of the curve shape. Finally, the influence was eliminated by the phase recovery method. The specific process was as follows:

Step 1: *G*_*j*_(*k*_*x*_, *k*_*y*_) was set to =  *S*′(*k*_*x*_, *k*_*y*_), where *S*′(*k*_*x*_, *k*_*y*_) was the original data with moving artifacts. The image was obtained by inverse Fourier transform, and the region of interest (ROI) was extracted.
Step 2: *g*_*j*_′(*x*, *y*) was set to = . All pixels outside the ROI of the image were forced to be zero gray, and *R* was the target area. *g*_*j*_(*x*, *y*) was conversed to frequency-domain rate, which could be expressed as *G*_*j*_′(*k*_*x*_, *k*_*y*_)=*F*{*g*_*j*_(*k*_*x*_, *k*_*y*_)} or as follows:
  (4) $$\begin{array}{c} g_{j}\left(k_{x},k_{y}\right)=\left\{\begin{array}{cc} g_{j}^{\prime }\left(x,y\right), & \left(x,y\right)\in R,\\ 0, & \text{others.} \end{array}\right) \end{array}$$
 Step 3: the phase of the data obtained in the previous step was taken, *ϕ*′(*k*_*x*_, *k*_*y*_) = arg[*G*_*j*_′(*k*_*x*_, *k*_*y*_)] − arg[*G*_*j*_(*k*_*x*_, *k*_*y*_)].
 Step 4: *G*_*j*+1_(*k*_*x*_, *k*_*y*_) was set to =  *e*^*iϕ*′(*k*_*x*_, *k*_*y*_)^|*S*′(*k*_*x*_, *k*_*y*_)|. Step 2 was repeated to iterate repeatedly until a satisfactory result was obtained.

### 2.3. Inspection Method

CT examination and MRI examination were performed on 71 patients, and the results were compared with those of laparotomy or laparoscopic surgery, so as to analyze the accuracy and application value of CT examination and MRI technology. Both MRI and CT examinations were taken in the supine position before the examination, and the patients were informed of the relevant precautions for the two groups of examination methods. During the examination, the patients had to follow the doctor's guidance and remove metal products such as earrings and necklaces in advance. During the scanning, the patients had to be calm, so as to eliminate the patient's own influence on the test results. The older patients had to be guided to get up and dress slowly and prevent falls. In addition, the impacts of psychological and economic factors of the patient on the examination results should be avoided as much as possible. MRI examination and CT examination were as follows.

MRI examination was performed with the MRI system scanning instrument (produced by PHILIPS company, 0.23 T long conduction type). The scan coefficient settings were defined as follows: T1WI (TR/TE was 440 ms/25 ms) and T2WI (TR/TE was 4000 ms/117 ms). Parameter settings were given as follows: the scan thickness was 6.0 mm, the interval thickness was 7.0 mm, and the matrix was 256 × 256.

CT examination was performed using the spiral CT ultrasound scanner (produced by GE). The scan parameters were given as follows: 120 kV, 220–300 mAs, slice thickness of 10 mm, spacing of 2 mm, and matrix of 256 × 256.

### 2.4. Research Indicators

The diagnosis results of 71 patients with FPA and the volume of mass in the pelvis were observed and recorded. The pathological results diagnosed by laparotomy or laparoscopic surgery were undertaken as the gold standard to compare the diagnostic accuracy of MRI and CT examinations in terms of disease types and mass size.

### 2.5. Statistical Analysis

The data obtained from the study were entered into SPSS 21.0 software for statistical analysis. The count data were given in the number of cases or percentages, and the chi-square test was adopted for comparison. The measurement data was given in the form of mean ± standard deviation, and the *t*-test was adopted for comparison. The test level was *α* = 0.05. When *P* < 0.05, the difference was considered to be statistically obvious, and *P* < 0.01 indicated that the difference was extremely obvious.

## 3. Results

### 3.1. Comparison between the Original MRI Image and the MRI Image after Artifact Elimination Based on Phase Correction Algorithm

**Figure 1:**
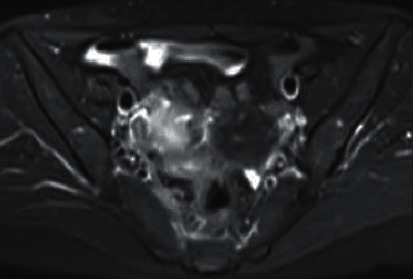
Original MRI image of an FPA patient.

**Figure 2:**
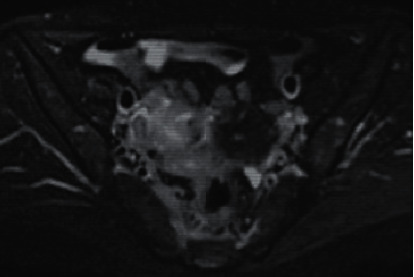
MRI image after artifact elimination based on phase correction algorithm.

In the steps to eliminate motion artifacts in MRI images, the most important thing was to extract the ROI of the image. The accuracy of extracting the ROI determined the effect of motion artifact elimination [13]. Initially, the ROI of the image was extracted manually, but such an extraction method would lead to inaccurate diagnosis results. Therefore, it was improved in this study, and a more advanced algorithm (level set algorithm) was adopted. Compared with the original algorithm, the ROI could be extracted more accurately, and the result of motion artifact removal was more accurate.  Figure 1 shows the original MRI image, and Figure 2 is the MRI image obtained after eliminating the motion artifacts by phase correction algorithm. It was clear intuitively that the artifact elimination for MRI based on the phase correction algorithm realized a good effect.

### 3.2. Comparison between Diagnosis Results of CT and MRI Examinations

**Figure 3:**
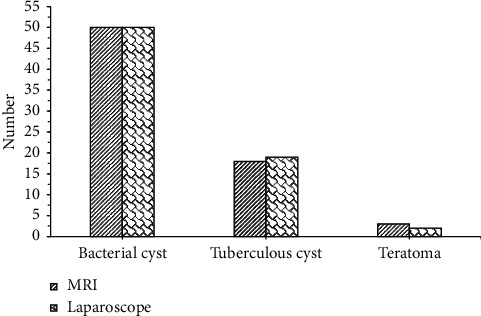
Comparison of diagnosis results between MRI and pathological examination.

**Figure 4:**
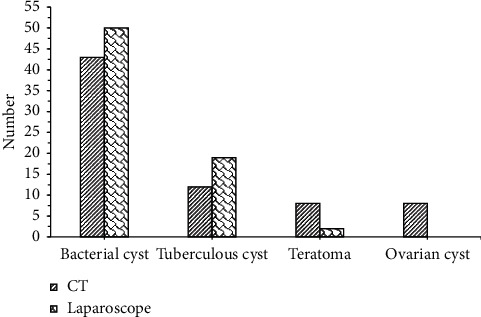
Comparison of the diagnosis results of CT examination and pathological examination.

The results of MRI examination are shown in Figure 3. Bacterial cysts (50 cases), tuberculous cysts (18 cases), and teratomas (3 cases) detected by MRI were similar to the results of laparoscopy (50 cases, 19 cases, and 2 cases), and there was no difference statistically significant (*P* > 0.05). The results of the CT examination are shown in Figure 4. There were bacterial cysts (43 cases), tuberculous cysts (12 cases), teratomas (8 cases), and ovarian cysts (8 cases) detected by CT, different from the results of laparoscopy (50 cases, 19 cases, 2 cases, and 0 cases), and the difference was statistically significant (*P* < 0.05). In addition, the patients with bacterial cysts (43 cases) and tuberculous cysts (12 cases) detected by CT were significantly lower than those of MRI (50 cases, 18 cases), and the difference was statistically significant (*P* < 0.05). Therefore, it was concluded that MRI examination was superior to CT examination in detecting bacterial cysts, which was the same as laparoscopic diagnosis. Tuberculous cyst examination was much better than CT examination, but slightly inferior to the laparoscopic diagnosis result. Because a case of tuberculous cyst was misdiagnosed as teratoma by the tuberculous cyst examination, CT examination mistakenly diagnosed 7 cases of bacterial cysts and 7 cases of tuberculous cysts as 6 cases of teratoma and 8 cases of ovarian cysts, respectively.

### 3.3. Comparison on Mass Volume Detected by CT and MRI Examinations

**Figure 5:**
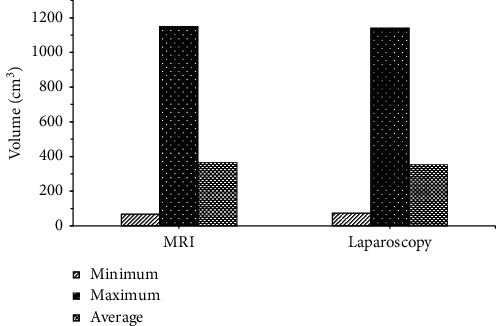
Comparison on mass volume between MRI and pathological examination.

**Figure 6:**
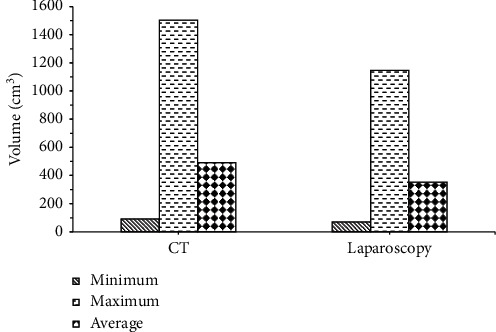
Comparison of mass volume between CT examination and pathological examination.

The minimum and maximum detected mass size of MRI was 4.1 cm × 4.2 cm × 3.9 cm and 13.9 cm × 9.5 cm × 8.7 cm, respectively. Spiral CT scan results showed that the detected mass size was 5.2 cm × 4.3 cm × 4.1 cm (minimum) and 15.5 cm × 10.1 cm × 9.6 cm (maximum), respectively. The comparison results showed that the mass of MRI examination (Figure 5) was smaller than that of CT examination (Figure 6), which was closer to the results of laparoscopic pathology.

### 3.4. Comparison on Images of CT and MRI Examinations

The MRI and CT images of the same patient were selected. The basic information of the patient was as follows: female, 45 years old, suffered from menopausal for 5 years, vaginal yellow discharge for 2 years, persistent lower abdominal pain without obvious cause for 20 days, and clay-colored stool; there was no fever, nausea, and vomiting; the urination was normal, the diet and sleep were normal, and weight loss was obvious. During the surgery, it was found that the rectum was adhered with surrounding tissues; about 5 mL of thick purulent fluid flowed out from the rectal recess; walls of the rectum and sigmoid colon showed edema and were thickened; and left ovary increased by about 5 cm × 3.5 cm × 4 cm. The postoperative diagnosis was pelvic abscess.

**Figure 7:**
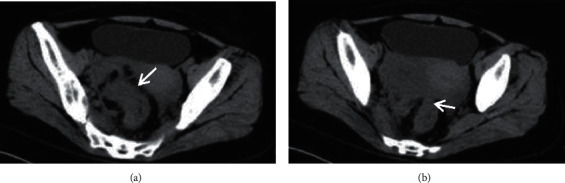
CT images of FPA patients. (a, b) CT plain scan images with a layer thickness of 10 mm and an interval of 2 mm, respectively. There were multiple cord adhesions and irregular mass in the pelvic cavity.

**Figure 8:**
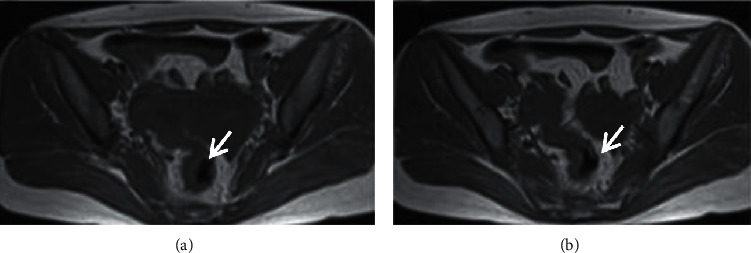
MRI images of FPA patients. (a, b) MRI plain scan images with a layer thickness of 6 mm and an interval of 7 mm, respectively. The fat borders were blurred, and the multiwinding shadows were visible.

Figure 7 (both Figures 7(a) and 7(b)) shows the CT examination images of patients. The CT image data showed that the irregular soft-tissue-like “mass” in the pelvic cavity was visible, with blurred edges and adhesion with surrounding tissues; the normal tissues and organs were difficult to identify; the density of the pelvic fat interstices was increased and blurred, and there were multiple cord adhesions and even “frozen pelvis,” and there was a little low-density effusion in the uterine rectum.  Figure 8 (both Figures 8(a) and 8(b)) shows the MRI images. The fat interface between the pelvic internal organs was blurred, and there were multiple cord adhesions, forming an inflammatory adhesion mass and even “frozen pelvis”; salpingitis with hydrops could be manifested as abscess-like masses between the uterine horns and ovaries, with low signal on T1WI and high signal on T2WI; the pus showed low signal on T1WI, high signal on T2WI, and high signal on DWI with a thick abscess wall; there was a little low-density effusion in the uterine rectal cavity. The comparison revealed that MRI could show the surrounding structure of pelvic abscess more clearly and display the specific conditions of the internal lesions of patients visually, so that medical workers could better determine the treatment and surgical plans based on the condition of the disease.

## 4. Discussion

Pelvic abscess is mostly caused by post-uterine-operation infection, poor sexual hygiene, lower reproductive tract infection, etc., which has a serious impact on the quality of life of patients [14]. Therefore, it is necessary to diagnose and treat as soon as possible. Doctors can make accurate diagnosis and identification of pelvic abscess by using MRI imaging methods, which is of great significance to promote the early recovery of the patient's disease [15, 16]. In order to eliminate the motion artifacts of MRI images and improve the clarity of the images, in this study, the MRI detection model was first established based on the phase correction algorithm, and the effect of the algorithm in eliminating the motion artifacts of MRI images was studied. The results found that compared with the original MRI image, it can be seen intuitively that the MRI artifact elimination based on the phase correction algorithm has a good effect. This is similar to the research results of Lo et al. [17], which improved the phase correction algorithm to improve the clarity of MRI images.

Afterwards, the phase correction algorithm was applied to the MRI diagnosis of 71 female patients with pelvic abscess admitted to our hospital and compared with the diagnosis results of multislice spiral CT and laparoscopy. The results showed that the bacterial cysts (50 cases), tuberculous cysts (18 cases), and teratomas (3 cases) detected by MRI were similar to the results of laparoscopy (50 cases, 19 cases, and 2 cases), and the difference was not statistically significant (*P* > 0.05); there were CT examination patients with bacterial cysts (43 cases), tuberculous cysts (12 cases), teratomas (8 cases), and ovarian cysts (8 cases), different from the results of laparoscopy (50 cases, 19 cases, 2 cases, and 0 cases), and the difference was statistically significant (*P* < 0.05). In addition, the CT examination results of bacterial cysts (43 cases) and tuberculous cysts (12 cases) were significantly lower than the MRI examination results (50 cases, 18 cases)), and the gap was statistically significant (*P* < 0.05). However, CT examination mistakenly diagnosed 7 cases of bacterial cysts and 7 cases of tuberculous cysts as 6 cases of teratomas and 8 cases of ovarian cysts. This is consistent with the results of Ma et al. [18]. In the study, the improved algorithm model was applied to MRI to diagnose female gynecological diseases, and the MRI examination results were significantly better than the CT examination results. The minimum size of the mass detected by MRI was 4.1  cm × 4.2  cm × 3.9 cm, and the maximum was 13.9  cm × 9.5  cm × 8.7 cm. The minimum size of the mass detected by the spiral CT scan was 5.2  cm × 4.3  cm × 4.1 cm, and the maximum was 5.2  cm × 4.3  cm × 4.1 cm. The value was 15.5  cm × 10.1  cm × 9.6 cm. The volume of the mass shown by MRI examination was smaller than that shown by CT examination. The difference between the two was statistically significant (*P* < 0.05), and it was closer to the results of laparoscopic pathology. This was consistent with the findings of Revzin et al. [19]. The diagnostic results of MRI technology on the mass of the mass were similar to the pathological tissues, and they were all low with more slices of spiral CT. It could further illustrate the advantages of using MRI technology in the imaging diagnosis of female pelvic abscess.

## 5. Conclusions

In this paper, MRI diagnosis of female pelvic abscess based on phase correction algorithm for MRI artifact elimination is carried out, and a comparative analysis is carried out with CT examination. The results show that the phase correction algorithm can eliminate the motion artifacts of MRI images and improve the clarity of the images. In the imaging diagnosis of female pelvic abscess, MRI diagnosis based on phase correction algorithm is more effective than multislice spiral CT diagnosis. But, there are also some deficiencies; the sample size studied in this paper is small, and the follow-up can expand the sample size and extend the content of the study. It is believed that, in the near future, MRI examination will show a brighter application prospect in the diagnosis of pelvic abscess with the continuous advancement of science and technology and the continuous development of imaging examination technology and equipment.

## Figures and Tables

**Table 1:** The main clinical manifestations of the 71 patients.

Clinical manifestations	High fever	Vaginal bleeding	Lower abdominal pain	Nausea and vomiting	Physical fatigue
Case	20	36	23	19	24

## Data Availability

The data used to support the findings of this study are available from the corresponding author upon request.
